# Interaction of Newcastle disease virus V protein with EFTUD2 modulates MDA5 pathway to suppress viral replication

**DOI:** 10.1016/j.psj.2025.105470

**Published:** 2025-06-24

**Authors:** Yin Han, Fan Zhang, Ziqing Zhou, Yufang Huang, Ruiai Chen

**Affiliations:** a College of Veterinary Medicine, South China Agricultural University, Guangzhou 510642, PR China; b Zhaoqing Branch Centre of Guangdong Laboratory for Lingnan Modern Agricultural Science and Technology, Zhaoqing 526238, PR China

**Keywords:** Newcastle disease virus, Chicken MDA5, Spliceosome factor, Elongation factor Tu GTP-binding domain-containing protein 2

## Abstract

Elongation factor Tu GTP-binding domain-containing protein 2 (**EFTUD2**), a core component of U5 small nuclear ribonucleoprotein particles (**snRNPs**), recently emerged as a novel innate immune regulator. Although the Newcastle disease virus (**NDV**) V protein facilitates immune evasion, interactions between NDV and spliceosome components remain poorly characterized. Using immunoprecipitation-mass spectrometry (**IP-MS**), we identified EFTUD2 as an interacting partner of the NDV V protein. Co-immunoprecipitation (**Co-IP**) and confocal microscopy confirmed this interaction, which we mapped primarily to residues 116–825 of EFTUD2. EFTUD2 overexpression enhanced chicken MDA5 (**chMDA5**) splicing efficiency, upregulated interferon-stimulated genes (**ISGs**) and interferon-β (**IFN-β**) production, and consequently suppressed NDV replication. Conversely, siRNA-mediated EFTUD2 knockdown promoted viral replication. Notably, EFTUD2’s regulation of chMDA5 splicing occurred specifically during NDV infection, suggesting that the V protein potentially activates EFTUD2’s splicing function. These findings establish EFTUD2 as a critical host restriction factor against NDV and provide novel mechanistic insights into viral immune evasion and host defense.

## Introduction

Newcastle disease (**ND**) is an acute, highly contagious viral infection caused by Newcastle disease virus (**NDV**). The virus infects over 240 bird species, primarily through direct contact with infected individuals (Dimitrov et al., 2016). First identified in Newcastle, England, and Indonesia in 1926, NDV causes severe economic losses in the global poultry industry (Dharmayanti et al., 2024). According to World Organisation for Animal Health (**OIE**) criteria, NDV strains are classified by pathogenicity into lentogenic, mesogenic, and velogenic types (Kang et al., 2016). Lentogenic strains exhibit low virulence, causing subclinical infections or mild respiratory or enteric signs. Mesogenic strains demonstrate intermediate virulence, resulting in respiratory infections with moderate mortality. Velogenic strains are highly virulent and subdivided into viscerotropic and neurotropic types. Viscerotropic velogenic strains induce hemorrhagic gastrointestinal ulcers, lymphoid depletion, and necrotic foci in the spleen, liver, and gut-associated lymphoid tissue (**GALT**). In contrast, neurotropic velogenic strains are characterized by neurological signs including dyspnea, depression, opisthotonos, head twisting, and paralysis (Zhang et al., 2023). Although mandatory vaccination has reduced ND outbreaks in recent years, the poultry industry remains threatened by emerging NDV genotypes and atypical epidemics (Butt et al., 2019).

NDV, an enveloped, single-stranded negative-sense RNA virus, belongs to the genus *Avian orthoavulavirus* 1 within the Paramyxoviridae family (Amarasinghe et al., 2019). Its approximately 15.2 kb genome encodes six structural proteins—nucleocapsid (**NP**), phosphoprotein (**P**), matrix (**M**), fusion (**F**), hemagglutinin-neuraminidase (**HN**), and polymerase (**L**)—along with two nonstructural proteins (**V** and **W**) (Mcginnes et al., 2010). The V and W proteins are produced through co-transcriptional mRNA editing of the P gene: V results from insertion of a single non-templated guanine residue (+1 frameshift), while W requires insertion of two guanines (Steward et al., 1993; Karsunke et al., 2019). Consequently, V and P share identical N-terminal domains (residues 1-133) but possess distinct C-termini. In wild-type viruses, V protein is generated from approximately 29 % of P gene transcripts (Mebatsion et al., 2001). Mounting evidence indicates that V protein determines host range restriction and subverts innate immunity (Park et al., 2003). For example, it recruits RNF5 to degrade MAVS, thereby inhibiting type I interferon production (Sun et al., 2019). V protein also targets TXNL1 and hnRNP H1 to regulate apoptosis and promote cell proliferation for viral replication (Chu et al., 2018; Tong et al., 2021). Thus, V protein serves multifunctional roles in infection.

The spliceosome—a large ribonucleoprotein complex comprising U1, U2, U4/U6, and U5 subunits—catalyzes alternative splicing (**AS**) events. This process is essential for regulating gene expression and generating proteomic diversity (Nilsen and Graveley, 2010). As a core component of U5 small nuclear ribonucleoprotein particles (**snRNPs**), elongation factor Tu GTP-binding domain-containing protein 2 (**EFTUD2**) functions as a GTPase that regulates RNA splicing and post-splicing complex disassembly (Lines et al., 2012). EFTUD2 also acts as an innate immune regulator by: (1) controlling cytokine expression (e.g., TNF-α and IL-6) downstream of NF-κB through MyD88 alternative splicing, and (2) modulating RIG-I and MDA5 expression to regulate RLR pathway signaling (De Arras et al., 2014; Zhu et al., 2015). Notably, multiple viruses interact with spliceosomal components during infection. For instance, the Dengue virus (**DENV**) NS5 protein binds U5 snRNP components CD2BP2 and DDX23, reducing host pre-mRNA splicing efficiency to facilitate viral replication (De Maio et al., 2016). Similarly, SARS-CoV-2 Nsp16 inhibits global mRNA splicing by targeting the mRNA recognition domains of U1 and U2 spliceosomal subunits (Banerjee et al., 2020). Although NDV is known to trigger host AS events (Liu et al., 2021), the role of EFTUD2 in NDV replication remains unverified.

In this study, we identified EFTUD2 as an interacting partner of the NDV V protein through co-immunoprecipitation coupled with mass spectrometry (**Co-IP/MS**) in DF-1 cells. Functional validation confirmed this interaction and demonstrated that overexpression of EFTUD2 suppresses NDV replication, whereas its knockdown enhances viral propagation. Further investigation revealed that EFTUD2 activates the MDA5 signaling pathway to regulate IFN-β production and interferon-stimulated gene (**ISG**) expression. Strikingly, this immunomodulatory function occurs exclusively upon NDV infection, indicating that the V protein is essential for triggering EFTUD2-mediated antiviral activity. These findings not only establish spliceosomal factors as novel restriction elements against NDV but also elucidate a distinct mechanism by which EFTUD2 counteracts viral evasion strategies.

## Materials and methods

### Cell lines, virus and viral infections

Human cervical cancer cells (**HeLa**), human embryonic kidney cells **(HEK-293T**), and chicken fibroblasts (**DF-1**) were maintained in Dulbecco′s Modified Eagle Medium (DMEM; Gibco, USA) or DMEM/F-12 (Gibco, USA) supplemented with 10 % fetal bovine serum (**FBS**), 2 mM l-glutamine, and 1 % penicillin-streptomycin at 37°C under 5 % CO₂. The NDV strain DHN3 was isolated in 2015 and characterized by our laboratory (Wang et al., 2020). For infection assays, DF-1 cells seeded in 6-well plates were infected with NDV at the specified multiplicity of infection (**MOI**). Following 1.5-hour viral adsorption at 37°C, cells were washed and cultured in fresh DMEM/F-12 medium containing 1 % bovine serum albumin (**BSA**) in a humidified 5 % CO₂ incubator.

### Construction of recombined plasmids

The pCMV-Flag, pCMV-Myc and pCAGGS-HA vectors were constructed and stored by our group. To generate pCMV-Myc-EFTUD2 and its truncated variants, target gene fragments were PCR-amplified from DF-1 cell cDNA using primers detailed in Table 1. These fragments were subsequently cloned into pCMV-Flag or pCAGGS-HA vectors via seamless assembly with the pEASY®-Basic Cloning Kit (TransGen Biotech, China). For pCMV-Flag-V construction, we followed an established protocol (Qiu et al., 2016). Briefly, the P gene open reading frame (**ORF**) was amplified by RT-PCR from DHN3-infected DF-1 cells. A non-templated G nucleotide was then introduced into the conserved RNA editing site through site-directed mutagenesis, followed by homologous recombination into the pCMV-Flag vector.

**Table 1:** Primers used for construction of plasmids.

Primers	Sequence (5′→3′)
pCMV-Myc-EFTUD2-F	gccatggaggcccgaattcggATGGACACTGACCTGTACGA
pCMV-Myc-EFTUD2-R	gcggccgcggtacctcgagTTACATGGGGTAGTTGAGAACGA
pCMV-Flag-V1-F	gataagtccggatccATGGCTACCTTTACAGATGC
pCMV-Flag-V1-R	CGACCATGGGCCCCTTTTTAGCA
pCMV-Flag-V2-F	TGCTAAAAAGGGGCCCATGGTCG
pCMV-Flag-V2-R	tgcagcccggggcggccgcTTACTTACCTTCTGAGATAATGC
pCAGGS-HA-EFTUD2_Δ1-115_-F	gttccagattacgctctcgagATGGATTTCCTGGCAGATCTGA
pCAGGS-HA-EFTUD2_Δ1-115_-R	ttggcagagggaaaaagatctTCACATGGGGTAGTTGAGAACGA
pCAGGS-HA-EFTUD2_Δ116-432_-F	gttccagattacgctctcgagATGGACACTGACCTGTACGATGAG
pCAGGS-HA-EFTUD2_Δ116-432_-R	ttggcagagggaaaaagatctTCACATGGGGTAGTTGAGAACGA
pCAGGS-HA-EFTUD2_Δ433-825_-F	gttccagattacgctctcgagATGGACACTGACCTGTACGATGAG
pCAGGS-HA-EFTUD2_Δ433-825_-R	ttggcagagggaaaaagatctTCACATGGGGTAGTTGAGAACGA
pCAGGS-HA-EFTUD2_Δ826-972_-F	gttccagattacgctctcgagATGGACACTGACCTGTACGATGAG
pCAGGS-HA-EFTUD2_Δ826-972_-R	ttggcagagggaaaaagatctTCAGGTGGCCATCAGGAAAG
pCAGGS-HA-EFTUD2_Δ116-972_-F	gttccagattacgctctcgagATGGACACTGACCTGTACGATGAG
pCAGGS-HA-EFTUD2_Δ116-972_-R	ttggcagagggaaaaagatctTCACTCATAGACGGTGACCGG
pCAGGS-HA-EFTUD2_Δ1-825_-F	gttccagattacgctctcgagCCTCGTCTGATGGAGCCCTAC
pCAGGS-HA-EFTUD2_Δ1-825_-R	ttggcagagggaaaaagatctTCACATGGGGTAGTTGAGAACGA
pCAGGS-HA-EFTUD2_Δ1-115,826-972_-F	gttccagattacgctctcgagATGGATTTCCTGGCAGATCTGA
pCAGGS-HA-EFTUD2_Δ1-115,826-972_-R	ttggcagagggaaaaagatctTCAAGGGGTGGCCATCAGG

The sequences with lower-case letter are homologous sequences to the selected vectors and the sequences with upper-case letter are the primer sequences for gene amplification.

### Analysis of potential proteins interacting with NDV V in host cells by IP-MS

Briefly, DF-1 cells were seeded in 10 cm cell culture dishes and transfected with either pCMV-Flag-V or control pCMV-Flag vector when reaching 70–80 % confluence. After 36-hour incubation at 37°C, cells were lysed in 1 mL NP-40 lysis buffer. Lysates were incubated overnight at 4°C with anti-Flag antibody-conjugated agarose beads. Immunoprecipitated complexes were analyzed by mass spectrometry (Sangon Biotech, Shanghai, China).

### Co-immunoprecipitation and western blotting

The interaction between EFTUD2 and V protein was validated by co-immunoprecipitation (**Co-IP**) and western blotting. HEK-293T cells seeded in six-well plates were transfected with the indicated plasmids for 48 h. Cells were lysed in NP-40 lysis buffer at 4°C for 30 min. After centrifugation, 40 μL of supernatant was reserved as Input, while the remaining lysate was incubated overnight at 4°C with anti-Flag affinity beads (Sigma-Aldrich, USA). Beads were washed five times with NP-40 buffer and proteins eluted by boiling in 2 × SDS sample buffer. Protein samples were resolved by SDS-PAGE and transferred to nitrocellulose membranes (Merck Millipore, USA). After 1-hour blocking with 5 % skim milk at room temperature, the membranes were incubated with Flag (TransGen Biotech, China), Myc (TransGen Biotech, China), HA (Abcam, USA), EFTUD2 (Abclonal, China), MDA5 (Abclonal, China), Mx1 (Abclonal, China), Tubulin (Abmart, China), β-Actin (Proteintech, China) and HN antibodies, which was prepared and stored in our laboratory (Han et al., 2023). After washing, membranes were incubated with horseradish peroxidase (**HRP**)-conjugated goat anti-mouse/rabbit IgG (1:5000) for 1 h at room temperature. Protein bands were visualized using ECL substrate (Beyotime, China) on an ImageQuant 800 system (Cytiva, USA).

### Confocal microscopy assays

DF-1 cells were seeded in glass-bottom confocal dishes (12-well format) and cultured for 24 h to form monolayers. Cells were co-transfected with pCMV-Flag-V and pCMV-Myc-EFTUD2 plasmids using Lipofectamine 3000 reagent (Invitrogen, USA) according to the manufacturer's instructions. After 36 h of transfection the cells were fixed using 4 % paraformaldehyde for 10 minutes at room temperature, permeabilized in 0.5 % Triton X-100 for 10 minutes, and blocked with QuickBlock™ Immunol Staining Blocking Buffer (Beyotime, China) for 30 minutes. For immunostaining, samples were incubated with mouse anti-Myc mAb and rabbit anti-Flag pAb at a dilution of 1:500 for 1 h at room temperature, followed by incubation with Alexa Fluor 488-conjugated anti-mouse IgG and Alexa Fluor 594-conjugated anti-rabbit IgG at a dilution of 1:1000 for 1 h. Nuclei were counterstained using DAPI for 5 minutes. Stained cells were observed using a confocal laser scanning microscope Stellaris 5 fluorescence microscope (Leica, Germany). All images were captured and processed using Leica Application Suite X (Leica Microsystems).

### RNA extraction and quantitative real-time RT-PCR

Total RNA was isolated using TRIzol™ Reagent (Invitrogen, USA) and reverse transcribed to cDNA with HiScript® III RT SuperMix (Vazyme, China) following the manufacturer's protocol. RT-qPCR was performed on a QuantStudio™ 3 System (Applied Biosystems, USA) using ChamQ Universal SYBR qPCR Master Mix (Vazyme, China) under standard cycling conditions: 95°C for 30 sec, followed by 40 cycles of 95°C for 10 sec and 60°C for 30 sec. Gene expression levels were normalized to GAPDH and calculated relative to untreated controls using the 2^-ΔΔCt^ method (Han et al., 2023). Primer sequences are listed in Table 2.

**Table 2:** Primer sequences for RT-qPCR.

primers	Sequence (5′−3′)
q-GAPDH-F	TTGACGTGCAGCAGGAACAC
q-GAPDH-R	ACCACTTGGACTTTGCCAGA
q-EFTUD2-F	GACCGCTCCTACGGCTTGT
q-EFTUD2-R	CGTCTTGGCTCCCACCTTT
q-IFN-β-F	CCAGCTCCTTCAGAATACGG
q-IFN-β-R	TGGCTGCTTGCTTCTTGTCC
mature-Mx1-F	GTCATTACTCGCTGTCCTC
mature-Mx1-R	TCTTGGGCTTTTCTTATTGCT
pre-Mx1-F	GACGCTCTACAACATTGCT
pre-Mx1-R	CCATCAGACTTGTGCTCCA
mature-ISG12-F	ACACTCCTCAGGCTTTACCAG
mature-ISG12-R	CTCCTTTGCCACCCATTGAGA
pre-ISG12-F	CAGCCATAAAATGAGTGGTT
pre-ISG12-R	TCAGCCAGGTTTATCACCAT
mature-ISG15-F	CTTGTGCAGCATCTGAAGTCC
mature-ISG15-R	ATCTGCACGTCCTTCTTACGG
pre-ISG15-F	GAGTGAATCAAAATCTAGCCT
pre-ISG15-R	CTGTTCTAGCACAATGACCA
NDV-NP-F	CAGCTCATGCGTTTATATCGG
NDV-NP-R	CGCAAAGCTCATCTGGTCA
q-chMDA5-F	ACCAGACAGGTGGCCGAGAA
q-chMDA5-R	ACCGTCTCTGTTCCCACGACT
pre-chMDA5-F	GTCGGTACAGATACTACTGC
pre-chMDA5-R	TCCTCACCTTTAGATGCCT
mature-chMDA5-F	AACGGAGGATTTGAAACAGCA
mature-chMDA5-R	GACACTTGCATCTCCTACAGC

### RNA interference assay

DF-1 cells were seeded in 12-well plates and transfected with gene-specific siRNAs using Lipofectamine RNAi MAX (Invitrogen, USA) at 60–70 % confluence, following the manufacturer's protocol. After 6 h of transfection, the medium was replaced with DMEM/F12 medium containing 2 % FBS and incubated for an additional 24 h. The sequences of the siRNAs used in this study are as follows: siRNA chicken EFTUD2 1#: CGCAGCCUCUCACUGAGCCUAUUAU; siRNA chicken EFTUD2 2#: CCCAAUAAGAAGAACAAGA; siRNA chicken EFTUD2 3#: GCCUCUCACUGAGCCUAUUAUUAAA. These siRNAs targeting EFTUD2 and siRNA negative control (**siRNA-NC**) were designed and synthesized by Tsingke Biotechnology (Beijing, China). To investigate knockdown efficiency, EFTUD2 mRNA levels were assessed by RT-qPCR and protein levels in DF-1 cells were determined by Western blotting using rabbit anti-EFTUD2 pAbs.

### Statistical analysis

Data are expressed as mean ± standard deviation (**SD**) of at least three independent experiments. Student's t-test and one-way ANOVA model statistical analyses were performed using GraphPad Prism version 8.0 software (GraphPad Software, Inc., USA). A p-value was considered statistically significant if it was less than or equal to 0.05.

## Results

### Bioinformatics analysis of host proteins interacting with NDV V proteins in DF-1 cells

**Fig. 1:**
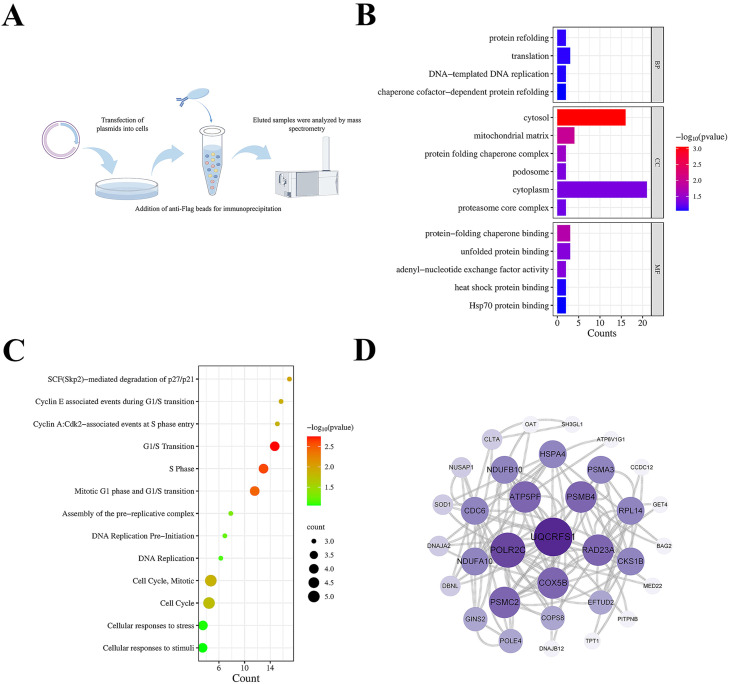
Bioinformatics analysis of host proteins interacting with NDV V proteins in DF-1 cells. (A) Schematic diagram showing the IP-MS procedure for mapping V-host protein interactomes. (B) Gene ontology analysis of cellular proteins interacting with V. (C) Reactome signaling pathway analysis of cellular proteins interacting with V. (D) The PPI network diagram of cellular proteins interacting with V.

To identify host proteins interacting with NDV V protein, we transfected DF-1 cells with Flag-tagged V expression constructs using empty vector as control. Thirty-six hours post-transfection, anti-Flag immunoprecipitation (**IP**) was performed, followed by mass spectrometry analysis of precipitated proteins to identify potential interactors (Fig. 1A).

Differentially interacting proteins were functionally annotated using the DAVID database. Gene Ontology (GO) enrichment analysis revealed three major functional categories, including biological processes, cellular components, and molecular functions. In terms of biological processes, proteins that interact with V are mainly involved in processes such as protein refolding, translation, and DNA-templated DNA replication (Fig. 1B). Differential proteins corresponding to significantly enriched molecular functions are mainly involved in protein-folding chaperone binding, unfolded protein binding, and adenyl-nucleotide exchange factor activity, suggesting that these proteins may be involved in the formation and release of viral particles (Fig. 1B). Moreover, to investigate the signaling pathways in which the identified proteins may be involved, we performed Reactome signaling pathway enrichment analysis. Among the categories, the differential proteins were mainly enriched in the pathways Cyclin E associated events during G1/S transition, G1/S Transition, and Mitotic G1 phase and G1/S transition, indicating that NDV V proteins may be associated with cell cycle alterations (Fig. 1C)(Tong et al., 2021).

To provide a comprehensive view of V-interacting proteins, we used the STRING database to analyze all protein interactions with V-interacting host proteins and visualized protein-protein interaction networks (PPI) using Cytoscape (Fig. 1D). Notably, EFTUD2 emerged as a high-confidence V-interacting partner. Subsequent investigations will delineate the EFTUD2-V interaction mechanism and its impact on viral replication.

### Verification of NDV V interaction with exogenous and endogenous EFTUD2

**Fig. 2:**
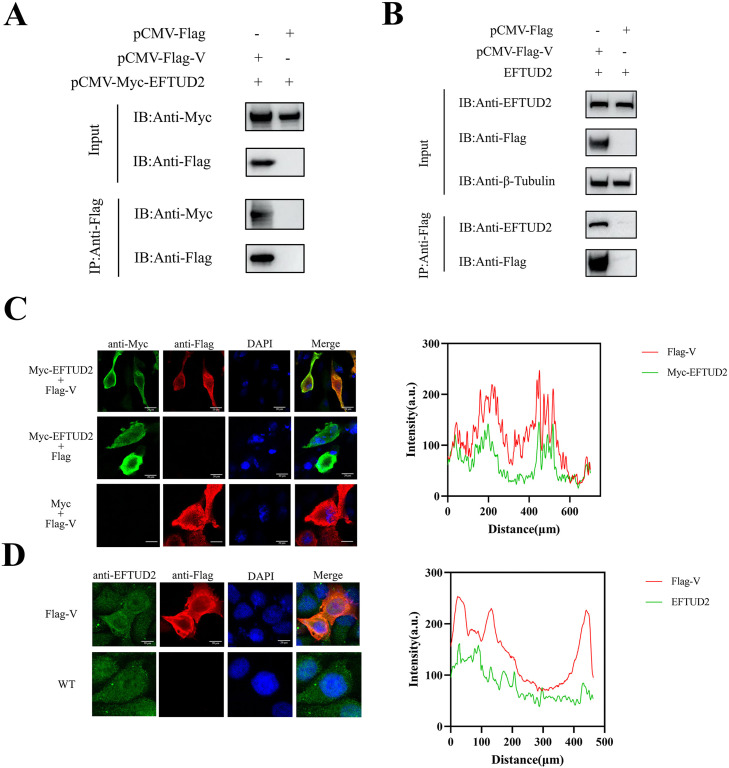
Verification of the interaction between V and exogenous and endogenous EFTUD2. (A) Interaction between Myc-EFTUD2 and Flag-V. HEK-293T cells transfected with pCMV-Myc-EFTUD2 and pCMV-Flag-V were immunoprecipitated using anti-Flag agarose beads and the precipitated proteins were subjected to Western blotting with anti-Myc and anti-Flag monoclonal antibodies. β-Tubulin was used as a control. (B) Interaction between Flag-V and the endogenous EFTUD2. HEK-293T cells transfected with pCMV-Flag-V and pCMV-Flag vector were immunoprecipitated using anti-Flag agarose beads, and the precipitated proteins were subjected to Western blotting with anti-EFTUD2 pAbs and anti-HA mAbs. β-Tubulin was used as a control. (C) The fluorescence co-localization analysis of Flag-V and Myc-EFTUD2 in plasmid co-transfected cells. DF-1 cells overexpressing Flag-V (red) and Myc-EFTUD2 (green) were immunostained with mouse anti-Myc monoclonal antibody and rabbit anti-Flag pAbs, respectively, and examined by confocal microscopy. Nuclei were stained with DAPI (blue). Measure of the fluorescence intensity of Flag-V and Myc-EFTUD2 at the same location. (D) Colocalization of NDV V with the endogenous EFTUD2 proteins in HeLa cells. Measure of the fluorescence intensity of Flag-V and EFTUD2 at the same location.

EFTUD2 was initially identified as a potential host interaction partner of the V protein through IP-MS. This interaction was subsequently confirmed in this study by CoIP. We constructed eukaryotic expression plasmids encoding Flag-tagged V (pCMV-Flag-V) and Myc-tagged EFTUD2 (pCMV-Myc-EFTUD2), alongside control plasmids (pCMV-Flag and pCMV-Myc-EFTUD2). Co-transfection of these plasmids into HEK-293T cells, followed by CoIP and Western blot analysis using anti-Myc and anti-Flag antibodies, demonstrated an interaction between V and exogenous EFTUD2 (Fig. 2A). To verify interaction with endogenous EFTUD2, immunoprecipitation assays were performed in DF-1 cells overexpressing Flag-tagged V. These assays confirmed the V-endogenous EFTUD2 interaction (Fig. 2B). Furthermore, confocal microscopy revealed co-localization of EFTUD2 and NDV V protein within the cytoplasm of DF-1 cells (Fig. 2C). Consistent results were obtained in HeLa cells, where exogenous NDV V co-localized with endogenous EFTUD2 in the cytoplasm (Fig. 2D). Collectively, these findings validate the interaction between the V protein and EFTUD2.

### EFTUD2 interacts with NDV V protein through the 116-825 amino acids domain

**Fig. 3:**
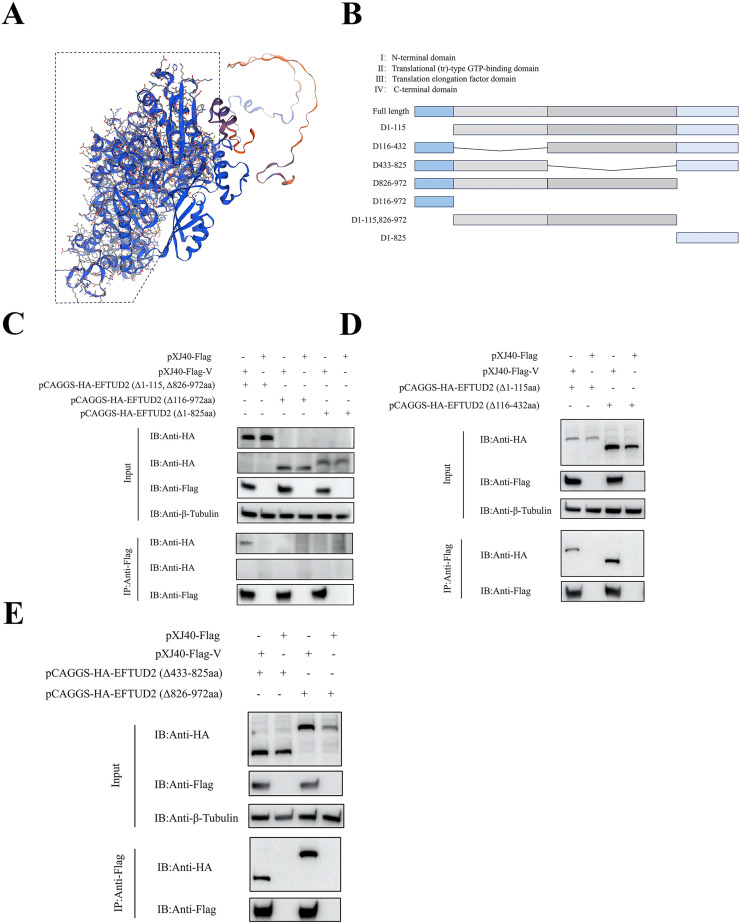
Identification and characterization of the key domain of EFTUD2-V interaction. (A) Homology modeling of chicken EFTUD2 protein using SWISS-MODEL. The dashed box in the figure shows the 116-825 amino acids region of the chicken EFTUD2 protein. (B) Schematic diagram illustrating the domain structures of EFTUD2 as well as truncated EFTUD2 constructs. The functional domains in EFTUD2 are shown with different colored boxes. (C, D, E) The 116-825 amino acids region of EFTUD2 are sufficient to allow a heterologous protein to associate with V. Multiple C-terminal and N-terminal truncations of HA-tagged EFTUD2 were expressed in DF-1 cells respectively. V were isolated by anti-Flag agarose beads, fractionated by SDS-PAGE, and analyzed by immunoblotting with the specific antibody.

To identify the EFTUD2 domain required for interaction with the NDV V protein, we generated a series of HA-tagged EFTUD2 truncation mutants based on its tertiary structure and confirmed their expression in DF-1 cells (Fig. 3A, B). Cells transfected with these mutants were harvested and subjected to immunoprecipitation using anti-Flag beads. The results showed that EFTUD2 N-terminal domain (EFTUD2_Δ116-972_) and C-terminal domain (EFTUD2_Δ1-825_) lost the ability to interact with NDV V protein (Fig. 3C).

To further delineate whether the entire EFTUD2 region spanning amino acids 116-825 or a sub-region mediates this interaction, we generated additional deletion mutants. Both EFTUD2Δ1-115 (N-terminal truncation) and EFTUD2Δ826-972 (C-terminal truncation) retained interaction with the V protein, corroborating our previous conclusion that neither the extreme N-terminus nor C-terminus is required (Fig. 3D, E). Furthermore, the double truncation mutant EFTUD2Δ1-115,826-972 also interacted with V. Crucially, both EFTUD2Δ116-432 and EFTUD2Δ433-825 interacted with the V protein (Fig. 3D, E). These findings indicate that the entire 116-825 region is involved in binding the V protein, and that interaction persists even if either segment (116-432 or 433-825) is individually missing. We conclude that the EFTUD2 structural domain spanning amino acids 116-825 is critical for interaction with the NDV V protein.

### Overexpression of EFTUD2 inhibits NDV replication

**Fig. 4:**
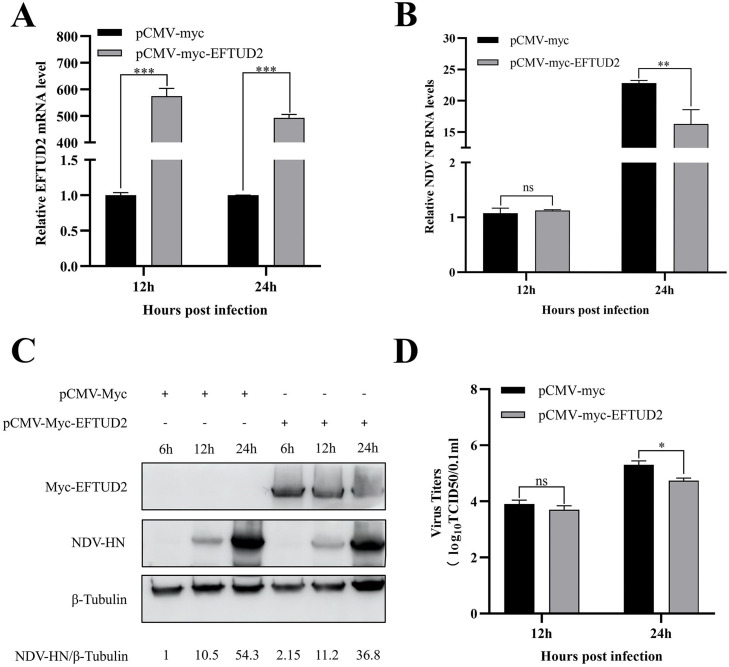
Overexpression of EFTUD2 inhibits NDV replication. (A) DF-1 cells were transfected with pCMV-Myc-EFTUD2 or pCMV-Myc vector for 24 h and infected with NDV (MOI=1). Cell samples were collected at different time points after viral infection. The mRNA levels of EFTUD2 were analyzed by RT-qPCR. (B) NDV-NP was determined by real-time PCR and homogenized using the chicken GAPDH gene as a control. (C) The protein levels of EFTUD2 and NDV-HN were detected by Western blotting in DF-1 cells. (D) The virus titers in the culture supernatant were determined by TCID50 assay. All results are presented as the mean ± SD of data from three independent experiments. *p < 0.05; **p < 0.01; ***p < 0.001; ns, *p* > 0.05.

To investigate the functional impact of the V-EFTUD2 interaction on NDV replication, we overexpressed EFTUD2 in DF-1 cells. Cells were transfected with either pCMV-Myc-EFTUD2 or the empty pCMV-Myc vector control, followed by infection with NDV at an MOI of 1. Samples were harvested at 6, 12, and 24 hours post-infection (**hpi**). The effect of EFTUD2 overexpression on viral replication was assessed using RT-qPCR, Western blotting and viral TCID50. EFTUD2 mRNA levels were significantly elevated in transfected cells compared to controls (Fig. 4A). RT-qPCR analysis demonstrated that NDV NP mRNA levels were significantly reduced at 24 hpi in EFTUD2-overexpressing cells relative to vector controls (Fig. 4B). Furthermore, Western blotting revealed significant suppression of NDV HN protein expression in the EFTUD2 overexpression group (Fig. 4C). Viral titers in supernatants were significantly decreased in EFTUD2-overexpressing cells at 24 hpi (Fig. 4D). Collectively, these results indicate that EFTUD2 overexpression inhibits NDV replication in DF-1 cells.

### siRNA-mediated knockdown of EFTUD2 promotes NDV replication

**Fig. 5:**
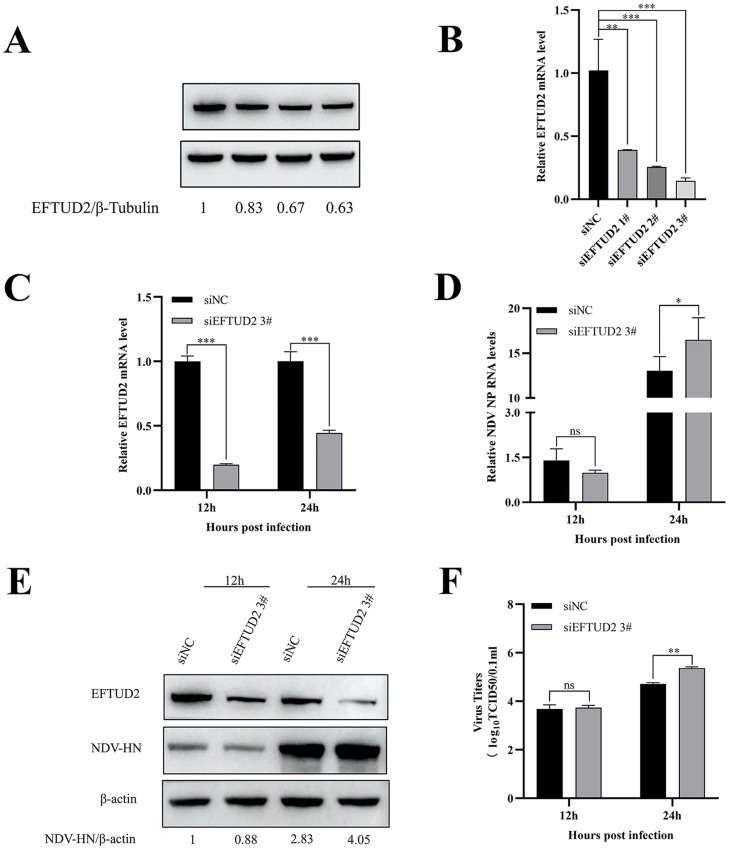
Knockdown of EFTUD2 inhibits NDV replication. (A) DF-1 cells were transfected with EFTUD2 siRNAs for 24 h. EFTUD2 protein expression was analyzed by Western blotting. (B) DF-1 cells were transfected with EFTUD2 siRNAs for 24 h. EFTUD2 mRNA levels were analyzed by RT-qPCR. (C) DF-1 cells were transfected with either siRNA EFTUD2 3# or siNC for 24 h, followed by infection with NDV (MOI=1). The expression of EFTUD2 mRNA was quantitated by real-time PCR. (D) NDV-NP was determined by real-time PCR and homogenized using the chicken GAPDH gene as a control. (E) DF-1 cells were transfected with either siRNA EFTUD2 3# or siNC for 24 h, and infected with NDV (MOI=1). The protein expression of EFTUD2 and NDV-HN were analyzed by Western blotting. The gray intensity for each band was measured, and the relative ratio of NDV HN was determined from the equation (Sample^HN^/Sample^actin^)/(Mock^HN^/Mock^actin^) and marked at the bottom of each lane. (F) The virus titers in the culture supernatant were determined by TCID50 assay. All results are presented as the mean ± SD of data from three independent experiments. *p < 0.05; **p < 0.01; ***p < 0.001; ns, *p* > 0.05.

To further elucidate the role of EFTUD2 in NDV infection, we performed siRNA-mediated knockdown of EFTUD2 expression. DF-1 cells were transfected with either EFTUD2-targeting siRNAs (siEFTUD2 1#, siEFTUD2 2#, and siEFTUD2 3#) or nontargeting control siRNA (**siNC**). Western blotting and RT-qPCR confirmed significant reduction of both EFTUD2 mRNA and protein levels in siRNA-transfected cells compared to siNC controls (Fig. 5A, B). Since siEFTUD2 #3 demonstrated the most efficient knockdown, it was selected for subsequent experiments.

To examine the impact of reducing endogenous EFTUD2 on NDV replication. DF-1 cells were similarly treated with siRNA and infected with NDV. The mRNA and protein levels of EFTUD2 were observed significant decrease in DF-1 cells treated with siEFTUD2 3#, compared to control cells (Fig. 5C, E). Following NDV challenge (MOI=1), EFTUD2 knockdown significantly increased NDV NP mRNA levels at 24 hpi (Fig. 5D). Correspondingly, Western blot analysis revealed elevated NDV HN protein expression in siEFTUD2 #3-treated cells compared to siNC controls (Fig. 5E). Viral titers were significantly enhanced in EFTUD2-knockdown cells at 24 hpi (Fig. 5F). These findings demonstrate that EFTUD2 depletion promotes NDV replication, suggesting its role as a negative regulator of viral infection.

### EFTUD2 positively regulates the MDA5/IFN-β signaling pathway

**Fig. 6:**
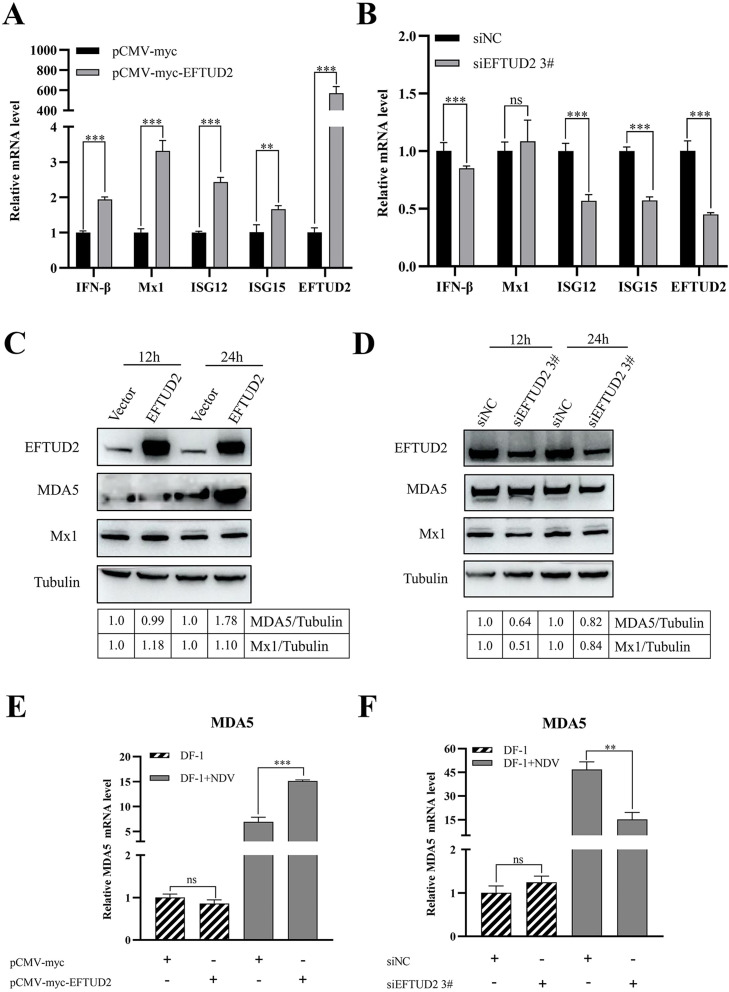
EFTUD2 positively regulates the MDA5/IFN-β signaling pathway. (A) pCMV-Myc-EFTUD2 or empty vector were transfected into DF-1 cells, and the cells were infected with NDV (MOI ​= ​1) at 24 ​h after transfection. Cells were harvested at 24 ​hpi, RT-qPCR was performed to determine the mRNA expression of vital ISGs, and IFNβ. (B) siEFTUD2 #3 or empty vector (siNC) were transfected into DF-1 cells, then the cells were infected with NDV (MOI ​= ​1) at 24 ​h after transfection. Cells were harvested at 24 ​hpi, RT-qPCR was performed to determine the mRNA expression of vital ISGs, and IFNβ. (C, D) DF-1 cells were transfected with pCMV-Myc-EFTUD2 or pCMV-Myc vector for 24 h or with an siRNA specifically targeting EFTUD2 (siEFTUD 3#) or siNC for 24 h before infection with NDV (MOI=1) or a mock control for another 24 h. The protein level of chMDA5, EFTUD2 and Mx1 was measured by western blotting. (E, F) DF-1 cells were transfected with pCMV-Myc-EFTUD2 or pCMV-Myc vector for 24 h or with an siRNA specifically targeting EFTUD2 (siEFTUD 3#) or siNC for 24 h before infection with NDV (MOI=1) or a mock control for another 24 h. mRNA expression of chMDA5 was measured by qPCR. All results are presented as the mean ± SD of data from three independent experiments. *p < 0.05; **p < 0.01; ***p < 0.001; ns, *p* > 0.05.

Type I interferon (**IFN-I**) constitutes the primary antiviral defense mechanism following viral infection. To determine whether EFTUD2 modulates IFN-β and interferon-stimulated gene (**ISG**) expression, DF-1 cells transfected with either pCMV-Myc-EFTUD2 or siEFTUD2 #3 were cultured for 24 h, then infected with NDV (MOI=1). RT-qPCR analysis at 24 hpi revealed that EFTUD2 overexpression significantly enhanced mRNA expression of IFN-β and key ISGs (including *Mx1, ISG12*, and *ISG15*), whereas knockdown suppressed these responses (Fig. 6A, B). This indicates that EFTUD2 positively regulates IFN-I signaling during NDV infection *in vitro*. Given established roles of EFTUD2 in innate immunity (De Arras et al., 2014), we examined chicken MDA5 (**chMDA5**) – a functional analogue of mammalian RIG-I in avian species (Lin et al., 2020). Consistent with prior findings (Zhu et al., 2015), EFTUD2 overexpression significantly increased chMDA5 protein and mRNA levels in NDV-infected DF-1 cells (Fig. 6C, E). Conversely, EFTUD2 knockdown reduced chMDA5 expression under infection conditions (Fig. 6D, F). Neither manipulation affected chMDA5 in uninfected cells (Fig. 6E, F). Since our earlier data establish direct EFTUD2-V interaction, we propose this interaction may mediate chMDA5 regulation during viral challenge.

### EFTUD2 regulates the splicing efficiency of chicken MDA5 by interacting with NDV V protein

**Fig. 7:**
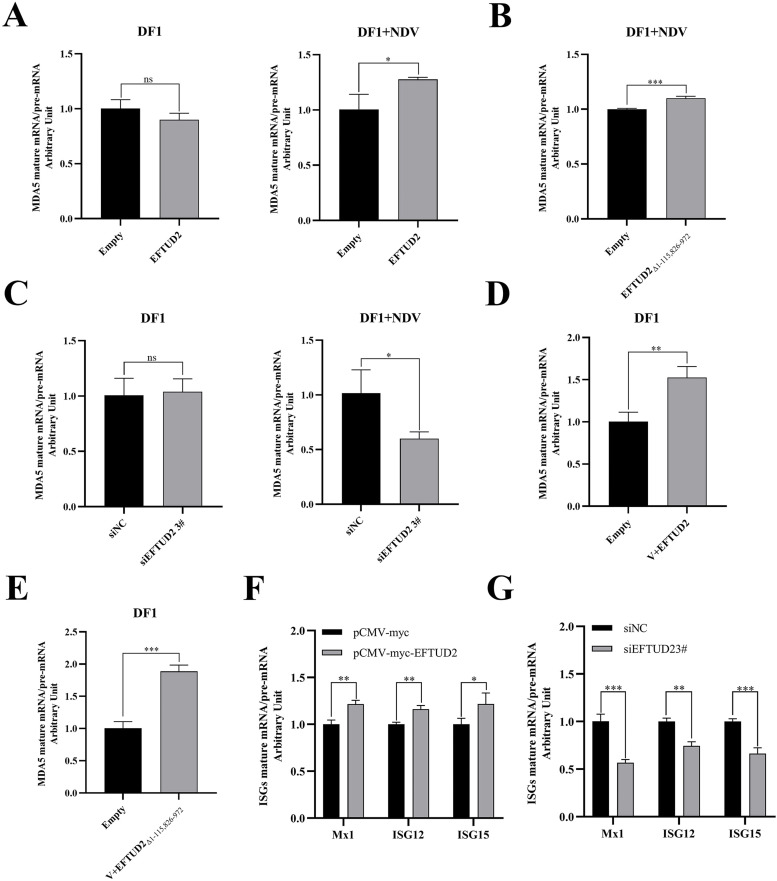
EFTUD2 regulates the splicing efficiency of chicken MDA5 by interacting with NDV V protein. (A, B and C) To evaluate the splicing efficiency of chMDA5, we designed special primers. One of the primers for detecting chMDA5 pre-mRNA is located within intron 4, and the primer for assessing chMDA5 mature-mRNA is located between exons 3 and 4. We found that overexpression of EFTUD2 (or EFTUD2_Δ1-115,826-972_) significantly increased the splicing efficiency of chMDA5, and knockdown of EFTUD2 significantly decreased the splicing efficiency of chMDA5. (D, E) Co-expression of V and EFTUD2 (or EFTUD2_Δ1-115,826-972_) in DF-1 cells significantly reduced the splicing efficiency of chMDA5 compared with an empty vector control. (F, G) Effect of overexpression or knockdown of EFTUD2 on the splicing efficiency of ISGs by RT-qPCR. All results are presented as the mean ± SD of data from three independent experiments. *p < 0.05; **p < 0.01; ***p < 0.001; ns, *p* > 0.05.

Given the established role of EFTUD2 as a core spliceosome component and its regulation of chMDA5 expression, we examined its impact on the maturation of endogenous chMDA5 pre-mRNA. chMDA5 pre-mRNA has an approximate length of 26.7 kb and contains 16 exons. Based on this, we focused on the excision of the intron between exons 3 and 4 to assess the splicing efficiency of chMDA5. To investigate the effect of EFTUD2 on chMDA5 mRNA processing, we designed special primers (Table 2) to assess the ratio between mature mRNA and pre-mRNA by RT-qPCR. Overexpression of EFTUD2 significantly increased chMDA5 splicing efficiency compared to controls, while EFTUD2 knockdown markedly reduced it (Fig. 7A, C). Notably, this regulatory effect was only observed under NDV infection conditions, suggesting EFTUD2 modulates chMDA5 splicing in association with viral infection. To determine whether NDV or specifically the NDV V protein mediated EFTUD2′s effect on splicing, we co-transfected pCMV-Flag-V and pCMV-Myc-EFTUD2 into DF-1 cells and measured chMDA5 splicing efficiency. As expected, co-transfection of V and EFTUD2 also significantly enhanced chMDA5 splicing compared to controls (Fig. 7D). Furthermore, transfection of a key EFTUD2 domain (aa 116–825), previously identified as the V-interacting region, yielded similar results, increasing chMDA5 splicing efficiency (Fig. 7B, E). To evaluate the specificity of EFTUD2′s effect, we also analyzed its impact on the splicing efficiency of ISGs. Consistent with our hypothesis, EFTUD2 overexpression enhanced ISG splicing efficiency, whereas knockdown impaired it (Fig. 7F, G). Collectively, these results support the model that EFTUD2 interacts with the NDV V protein to influence chMDA5 expression levels by regulating its pre-mRNA splicing efficiency.

## Discussion

NDV is a highly contagious avian pathogen affecting poultry and wild birds, characterized by high morbidity and mortality rates (Sun et al., 2022). While exhibiting zoonotic potential, human infections typically manifest as conjunctivitis (Jia et al., 2022). The NDV V protein is a well-established virulence factor that facilitates innate immune evasion in host cells (Audsley and Moseley, 2013). To elucidate V protein-host interactions, we employed immunoprecipitation-mass spectrometry to identify potential binding partners. Bioinformatics analysis revealed these interactors participate in NDV-related biological processes, enabling construction of a V-host protein interaction network. Within this network, EFTUD2 emerged as a key V-interacting protein, with the binding region mapped to EFTUD2 residues 116–825. Notably, EFTUD2 overexpression suppressed NDV replication, suggesting its role as a novel restriction factor in antiviral defense.

Newcastle disease virus, like other paramyxoviruses, potently induces type I interferon (IFN-β) production, a crucial mediator of host innate immunity (Seth et al., 2005; Kato et al., 2006). However, accumulating evidence demonstrates that NDV employs multiple strategies to evade innate immune recognition and signaling (Motz et al., 2013; Qiu et al., 2016). The NDV V protein is a key immune evasion factor, known to suppress RLR-mediated interferon signaling by targeting host proteins such as MDA5, LGP2, and STAT1 (Ulane et al., 2005; Childs et al., 2012). Additionally, V protein interacts with hnRNP H1 to regulate cell proliferation and enhance viral replication (Tong et al., 2021), consistent with our bioinformatic analysis of V-interacting proteins and their enrichment in relevant biological processes (Fig. 1C). Despite these advances, interactions between NDV and the cellular spliceosome remain poorly characterized. This large, dynamic ribonucleoprotein complex, comprising five snRNPs (U1, U2, U4/U6, U5) and numerous non-snRNP factors (Papasaikas and Valcárcel, 2016), catalyzes pre-mRNA splicing—a process critical for diverse biological functions and frequently exploited by viruses. For instance, Human cytomegalovirus (**HCMV**) infection significantly up-regulates the expression of the cellular RNA-binding protein CPEB1, leading to an overall shortening of the 3′ untranslated region and a lengthening of poly(A) tails in host transcripts(Batra et al., 2016). The M1, M2 and M3 proteins of vesicular stomatitis virus (**VSV**) induce cytoplasmic relocalization of hnRNPs, which inhibits host gene transcription (Redondo et al., 2015). Notably, recent work indicates NDV infection also triggers widespread alternative splicing events, particularly affecting genes involved in splicing regulation and immune responses, highlighting its significant impact on host pre-mRNA processing and immune gene expression (Liu et al., 2021).

EFTUD2 is a GTPase that regulates pre-mRNA splicing. It was previously identified as a novel innate immune regulator (De Arras et al., 2014). Multiple spliceosomal components, including EFTUD2, participate in interferon signaling pathways (Patzina et al., 2017). For example, cytoplasmic EFTUD2 and SNRNP200 act as RNA sensors that induce interferon production during viral infection (Tremblay et al., 2016; Boudreault et al., 2022). Hosts may evade antiviral responses through EFTUD2 downregulation, as observed in hepatitis C virus (HCV) infection where reduced EFTUD2 expression impairs RIG-I and MDA5 pre-mRNA splicing (Zhu et al., 2015). In this study, we demonstrate that the NDV V protein can interact with both exogenous and endogenous EFTUD2, with co-localization observed in the cytoplasm (Fig. 2C). The identification of components of U5 snRNP as interactors of the NDV V protein seems surprising because these cellular components are mainly located in the nucleus, but NDV replicates mainly in the cytoplasm. However, some examples exist of cytoplasmic viral proteins interacting with components of U5 snRNP, such as the dengue virus NS5 protein, which interacts with CD2BP2 and DDX23 of U5 snRNP to regulate AS within the host cell (De Maio et al., 2016). The RNA-dependent RNA polymerase (**RdRp**) of picornaviruses interacts directly with PRP8 to block pre-mRNA splicing and mRNA synthesis (Liu et al., 2014). Our results are in agreement with previous studies that the reovirus μ2 protein also co-localizes with the U5 snRNP component in the cytoplasm. A plausible explanation that can be given with reference to Boudreault et al. (Boudreault et al., 2022) is that the V proteins may interact with EFTUD2 in the cytoplasm, and then they enter into the nucleus to assemble to form functional snRNPs.

Previous studies have shown that NDV infection can lead to upregulation of chMDA5 (Fig. 6C, D), consistent with our results (Shao et al., 2023). EFTUD2 comprises an N-terminal domain, a GTP-binding domain, a translation elongation factor (**TEF**) domain, and a C-terminal domain. Its activation occurs upon GTP binding to the GTP-binding domain. Activated EFTUD2 then incorporates into U5 snRNP, assembling into a complex capable of splicing pre-mRNA. Consequently, the GTP-binding domain is essential for EFTUD2 function (Jia et al., 2020). Our findings show that the NDV V protein interacts with EFTUD2 within amino acids 116-825, a region covering both the GTP-binding and TEF domains. Given that this interaction overlaps the GTP-binding domain critical for splicing activity, we investigated whether EFTUD2′s regulation of chMDA5 involves its pre-mRNA splicing function. To assess host mRNA splicing efficiency, we designed specific primers spanning exon-exon junctions and within introns. Using RT-qPCR, we quantified mature mRNA and pre-mRNA levels, calculating the ratio of mature to pre-mRNA as a measure of splicing efficiency. This study revealed that EFTUD2 overexpression enhanced the splicing efficiency of chMDA5 and ISG pre-mRNAs. This led to upregulated expression of downstream type I interferons and ISGs, thereby inhibiting viral replication. Conversely, EFTUD2 knockdown reduced splicing efficiency and yielded the opposite effects. Collectively, these findings establish EFTUD2 as a restriction factor that negatively regulates NDV replication.

**Fig. 8:**
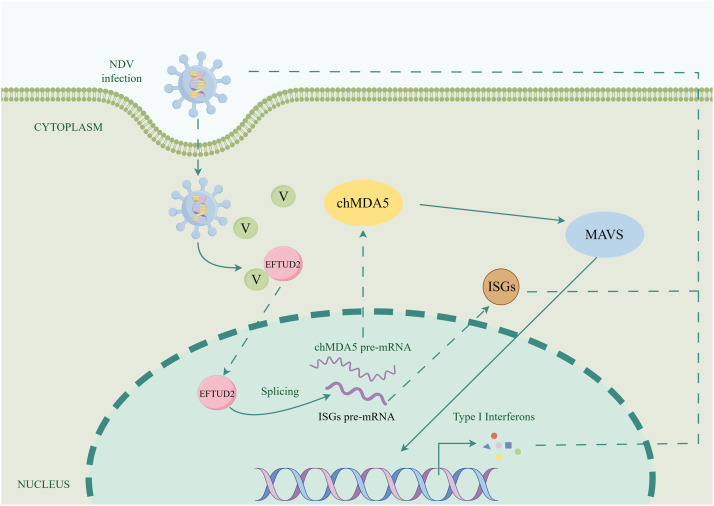
A proposed model for EFTUD2 in inhibition of NDV infection. (By Figdraw). Interaction between EFTUD2 and NDV V protein positively regulates the splicing efficiency of chMDA5 and ISGs, which induces downstream ISGs and IFN-β production to inhibit viral replication.

Based on our findings, we propose a model for EFTUD2 in innate immune regulation (Fig. 8), in which EFTUD2 exerts its antiviral effects by interacting with the NDV V protein to regulate the expression of chMDA5 through splicing and induce the expression of type I interferon.

In summary, this study demonstrates that the NDV V protein interacts with EFTUD2 to regulate chMDA5 and ISG splicing efficiency, thereby modulating IFN-β and ISG expression. These findings establish EFTUD2 as a host restriction factor that suppresses NDV replication. Our work advances understanding of spliceosome-mediated antiviral immunity and identifies EFTUD2 as a potential therapeutic target. Future studies should investigate whether similar mechanisms operate in other paramyxoviruses and evaluate EFTUD2 function *in vivo*.

## Disclosures

The authors declare no conflicts of interest.

## Declaration of competing interest

The authors declare that they have no known competing financial interests or personal relationships that could have appeared to influence the work reported in this paper.
